# Transfixed by transgenics: how pathology assumptions are slowing progress in Alzheimer's disease and related dementia research

**DOI:** 10.15252/emmm.202318479

**Published:** 2023-09-26

**Authors:** Luciano D'Adamio

**Affiliations:** ^1^ Department of Pharmacology, Physiology & Neuroscience New Jersey Medical School, Brain Health Institute, Jacqueline Krieger Klein Center in Alzheimer's Disease and Neurodegeneration Research, Rutgers The State University of New Jersey Newark NJ USA

**Keywords:** Genetics, Gene Therapy & Genetic Disease, Methods & Resources, Neuroscience

## Abstract

Model organisms of human diseases are invaluable tools for unraveling pathogenic mechanisms, identifying potential targets for drug development, and evaluating the therapeutic efficacy of candidates in preclinical trials. The utility of model organisms hinges upon their ability to faithfully replicate the underlying pathogenic mechanisms of the human disease. For rodent models of Alzheimer's disease (AD) and AD‐related dementias (ADRD), the limited translatability to human disease raises concerns about their overall utility. What factors contribute to this limitation? Is AD inherently too complex to be accurately modeled in nonhumans? Is the divergence between rodent brains and the human brain so pronounced that rodents are unsuitable as model organisms for AD? Or is it plausible that the commonly used rodent models don't capture the genuine pathogenic mechanisms underlying these diseases? This editorial discusses the challenges associated with transgenic models of AD and ADRD and offers some alternative approaches.

## Graphical abstract

In this commentary, Luciano D'Adamio discusses the challenges associated with animal models of Alzheimer's disease and related dementias that heavily rely on random insertion of presumed disease‐inducing factors.

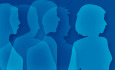

Rodent models for AD and ADRD have been meticulously selected to replicate the brain pathology characterizing these disorders. Overexpression of pathogenic mutants from genes responsible for early‐onset familial forms of AD/ADRD effectively reproduces certain desired pathology, notably amyloid plaques and, in some cases, neurofibrillary tangles (NFTs), neuronal loss, and neuroinflammation. Among the frequently employed genes are *APP* (encodes the precursor of the major Amyloid plaque component, Amyloid‐β (Aβ)), *PSEN1* and *PSEN2* (encoding the gamma‐secretase catalytic component, an essential protease for Aβ production from APP), and *MAPT* (whose protein product tau is the primary component of NFTs). These transgenic (TG) models are generated by random insertion of one or more minigenes expressing pathogenic mutants, regulated by promoters with robust activity in neurons. While AD and ADRD pathology typically develops over several decades in humans, TG models can manifest pathology within a matter of months, generally assumed to provide an accelerated representation of the disease process. The notion that similar pathology critically determines the relevance of an AD or ADRD model is rooted in the assumption that this pathology represents the primary pathogenic factor in these diseases. In this pathogenic framework, TG models have yielded valuable insights into mechanisms driving amyloid pathology and the interplay between amyloid and tau pathologies. Notably, three amyloid‐targeting antibodies recently approved by the FDA underwent preclinical trials in TG models. In‐depth discussion on these aspects can be found in various informative reviews (Sasaguri *et al*, 2017; Dawson *et al*, 2018).

Yet, TG models have limitations that can negatively influence experimental outcomes. The major risks associated with the TG approach primarily arise from three key factors: (i) Quantitative, qualitative (cell‐type specificity), spatial, and temporal patterns of “disease gene” expression are not controllable and often divergent to human expression. (ii) Overexpression of hypomorphic mutations, which lead to disease through partial loss‐of‐function but may paradoxically display a gain‐of‐function phenotype. (iii) Insertion/deletion (TgINDEL) mutations and chromatin modifications resulting from the random insertion of transgenes. Next, persuasive data that vividly illustrate these confounding effects in TG models are discussed. Some of these also provide compelling evidence that cognitive impairment following APP overexpression is largely independent of Aβ and amyloid pathology. Yet, these models continue to be extensively utilized to study the pathogenic role of Aβ and amyloid and assess the therapeutic intervention efficacy in preclinical studies.

APPsi: tTA mice are TG animals expressing a tetracycline transactivator (tTA) driven by the CaMKIIα promoter and a chimeric mouse/human APP with the pathogenic Swedish and Indiana mutations regulated by a tetracycline‐responsive promoter. Neuronal overexpression of the mutant APP can be suppressed by doxycycline. These mice exhibit characteristic features observed in other TG AD models, including locomotor hyperactivity, cognitive impairments, amyloidosis, elevated levels of soluble and oligomeric Aβ, as well as an epileptiform phenotype with network hyperexcitability. Delaying transgene APP expression onset until adulthood can notably alleviate locomotor hyperactivity, highlighting how overexpression of mutant APP during early postnatal development leads to persistent developmental changes in motor circuits and pronounced locomotor hyperactivity (Rodgers *et al*, 2012). When mutant APP expression is suppressed after the onset of amyloid pathology and cognitive deficits, a rapid improvement in cognitive performance is observed, accompanied by a large decrease in levels of full‐length APP, soluble APP ectodomains, and APP C‐terminal fragments. However, amyloid deposits and pools of soluble, insoluble, and oligomeric Aβ42 remain (Melnikova *et al*, 2013). Another study demonstrated that reduction of Aβ using a γ‐secretase inhibitor had no effect on network hyperexcitability in this animal model, whereas it was effectively normalized by suppressing transgene expression (Born *et al*, 2014). These studies suggest that transgenic overexpression of APP, soluble APP ectodomains, and/or APP C‐terminal fragments may play a more significant role in mediating cognitive deficits and other abnormalities in these mice compared with Aβ overproduction and amyloid pathology. Given that TG models reliant on APP overexpression exhibit elevated levels of APP and its metabolites, it is reasonable to infer that in these models, cognitive deficits and other abnormalities could predominantly stem from increased expression of APP, soluble APP ectodomains, and/or APP C‐terminal fragments, rather than being primarily driven by Aβ oligomers and amyloid pathology.

To investigate the direct role of Aβ in cognitive impairment, Kim et al examined cognition in BRI2‐Aβ transgenic mice that produce Aβ40, Aβ42, or both peptides from a transgenic BRI2‐Aβ fusion protein (McGowan *et al*, 2005). Despite high Aβ levels, Aβ oligomers and amyloid pathology, the BRI2‐Aβ mice have intact cognitive performance, providing evidence that Aβ and amyloid pathology alone do not impair cognition and that other APP processing derivatives or APP overexpression or misexpression may play a major role in the APP TG models' cognitive decline (Kim *et al*, 2013). Indeed, these findings from the BRI2‐Aβ model align with the observation that there are individuals with amyloid pathology that do not display cognitive impairments typical of AD.

Neuronal cell‐specific promoters used in AD/ADRD TG models may overlook critical pathogenic mechanisms involving non‐neuronal cell types, like microglia, which are known to play a significant role in the disease. For example, PSEN1/2 in microglia are involved in the processing of TREM2, a microglia‐specific protein associated with sporadic AD. The possibility that pathogenic PSEN1/2 mutations could alter TREM2 processing/function, contributing to the pathogenic mechanisms associated with PSEN1 mutations, raises concerns about potential oversight of this pathogenic pathway in such TG models.

The rTg4510 mouse model of tauopathy exemplifies the significant impact of TgINDEL mutations. This model has been extensively used in basic and preclinical studies for over a decade, mainly due to its pronounced neurodegeneration and NFT pathology. rTg4510 mice overexpress a mutant tau variant (tauP301L) associated with frontotemporal dementia and the tTA required to drive expression from the tauP301L transgene. rTg4510 mice were produced by pronuclear injection of minigene constructs into mouse zygotes, which produced various random integration events. The rTg4510 line was chosen for its robust NFT pathology and neurodegeneration, and none of the selected mutations were mapped or characterized. Recent work showed that the premature neurodegeneration and NFTs observed in rTg4510 mice do not solely arise from the overexpression of tauP301L, but rather from unintended dysfunctions induced by the tauP301L and tTA TgINDEL mutations. In particular, the rTg4510 tauopathy‐like phenotype requires a ~70‐copy tau‐transgene insertion causing a 244‐kb deletion in the *Fgf14* gene locus and a ~7‐copy tTA‐transgene insertion causing a 508‐kb deletion that disrupts another five genes (Gamache *et al*, 2019). This example emphasizes the inherent risk of non‐disease‐related insertional effects when selecting TG lines based on a desired phenotype alone. Given that existing TG models of AD/ADRD either do not identify their integration site or do not analyze the potential contribution of TgINDEL mutations to the desired phenotype, there are significant limitations and untested confounding factors that make them potentially misleading for studying disease mechanisms and treatments.

The problematic issues with the TG approach could be mitigated by analyzing several experimental lines (e.g., TG with pathogenic mutations) and control lines (e.g., TG without pathogenic mutation) with comparable levels of transgene overexpression. After this initial screening, a few lines (≥3 lines) with phenotypes that represent the average characteristics observed in the larger pool of lines, alongside control lines, should be selected for follow‐up experiments. Lines with outlier phenotypes should be excluded as they are almost invariably due to TgINDEL mutations, at least until proven otherwise. This approach is time‐consuming and costly and will not eliminate confounding effects caused by overexpression.

In contrast, the knock‐in approach (KI) involves precise insertion of pathogenic mutations into rodent genes through gene editing, faithfully mimicking the genetic makeup of the disease. KI models do not rely on preconceived assumptions about pathogenic mechanisms. In KI models, endogenous regulatory elements control mutant gene expression, ensuring that disease‐related proteins are expressed in a physiologically relevant quantitative, cell‐specific, spatial, and temporal manner. Confounding effects of TgINDEL mutations are also avoided and at first glance, rodent KI models appear to offer only advantages without drawbacks.

Because KI models do not involve heightened APP expression, they circumvent the potential impacts on memory and synaptic transmission arising from overexpression of APP and its metabolites (Melnikova *et al*, 2013; Born *et al*, 2014). However, this same reason also hampers their ability to replicate brain pathology. Therefore, KIs face limited acceptance as AD/ADRD models due to the criterion of pathology validation. This dogmatic mindset, accepting genetically artifactual models while dismissing genetically faithful ones, appears counterintuitive. This rigid perspective is even more perplexing, considering that the outcomes of ~20 years of clinical trials targeting Aβ/amyloid, including the controversial FDA approval of antiamyloid antibodies, have not offered compelling evidence to substantiate a pivotal role of Aβ and amyloid pathology in AD/ADRD pathogenesis.

Among the few KI models gaining traction, the *APP* ^*NL‐G‐F*^ KI mice developed by Saito's laboratory stand out (Saito *et al*, 2014). This model incorporates humanized Aβ region with the Swedish “NL,” Iberian “F,” and Arctic “G” mutations into the mouse *App* allele. It recapitulates several pathologies, including amyloid plaques, synaptic loss, microgliosis, and astrocytosis. The success of this chimeric *APP* ^*NL‐G‐F*^ model stems from the fact that it shows early pathology through increased total Aβ production (Swedish mutation), elevated Aβ42/Aβ40 ratio (Iberian mutation), and facilitated Aβ aggregation with reduced proteolytic degradation (Arctic mutation). The advantages of this model over TG mice were discussed in a previous review (Sasaguri *et al*, 2017). Unfortunately, to align with the amyloid hypothesis‐based validation requirements, a chimeric *APP* allele packing three independent pathogenic mutations was used. This chimeric allele does not mimic naturally occurring pathogenic mutations and may lead to confounding effects if these mutations contribute to neurodegeneration through different, and potentially contrasting, mechanisms.

To faithfully represent human disease through unbiased human genetic evidence, our research group developed some of the initial mouse KI models of AD/ADRD, including a KI model of Familial Danish dementia (FDD). Familial Danish dementia (FDD) is a progressive neurodegenerative disease with cerebral amyloid plaques, neuroinflammation, and NFTs. FDD pathology is indistinguishable from AD pathology, and these cases were initially classified as familial AD forms. Familial Danish dementia (FDD) is caused by a mutation in *ITM2B* (Vidal *et al*, 2000), a gene that encodes a type II membrane protein called BRI2. BRI2 is synthesized as a precursor protein cleaved at the C terminus in the trans‐Golgi by proprotein convertase into mature BRI2 and a 23 amino acid‐long (Bri23) soluble C‐terminal fragment. The FDD *ITM2B* mutation changes the BRI2 C‐terminal region, producing a longer C‐terminal fragment, which is processed into an amyloidogenic peptide (Adan) while leaving mature BRI2 unchanged. In patients with FDD, amyloid plaques are formed by Aβ and ADan, either alone or in combination (Vidal *et al*, 2000). Due to these differences in plaque composition, FDD has been reclassified as an ADRD.

In FDD KI mice, the levels of mature BRI2 in critical cellular compartments, including synapses, are diminished due to the partial targeting of the FDD mutant BRI2 immature protein for Endoplasmic Reticulum‐associated degradation (ERAD; Yin *et al*, 2021a, 2021b). A fraction of immature mutant BRI2 escapes ERAD and is processed into ADan, unique to individuals with the FDD mutation, and mature BRI2. This partial loss of mature BRI2 results in impaired synaptic plasticity, learning, and memory deficits—similar to those observed in BRI2 haploinsufficient mice—without detectable ADan deposition (Tamayev *et al*, 2010). These findings challenge the notion that ADan and AD‐like pathology solely drive neuronal damage and dementia in individuals with FDD and indicate that the FDD mutation behaves like a hypomorphic mutation with reduced levels of functional mature BRI2 while also generating the mutant, pro‐amyloidogenic ADan peptide. Possibly, the aggregation tendency of the mutant COOH‐terminal sequences may contribute to both phenotypes: causing misfolding of the mutant precursor BRI2 protein in the ER, leading to ERAD, and promoting the amyloid properties of ADan. These combined effects may play a role in the complex pathogenesis of FDD.

The evidence of learning and memory deficits occurring in this genetically faithful model of FDD, independent of “pathology” and with a significant loss‐of‐function component, had the potential to provoke a paradigm shift in the field of AD/ADRD research. But this model has been largely dismissed as inadequate for modeling human FDD due to its failure to meet the pathology‐focused validation requirements. This dismissal has raised doubts about the relevance of other phenotypes, such as the hypomorphic behavior of the FDD KI mutation, in relation to the human disease. Yet, the FDD KI model finds support in several aspects. Analysis of human brain samples from FDD patients reveals reduced levels of mature BRI2 and detectable levels of immature FDD mutant BRI2, mirroring the findings in the FDD KI mice. This biochemical consistency supports the notion that a hypomorphic pathogenic mechanism may be at play in both human FDD and the mouse KI model. Moreover, BRI2's diverse functions, from its involvement in synaptic transmission, APP processing, and antiamyloidogenic effects to its significant expression in microglia and interaction with TREM2 processing, establish clear connections between BRI2 and crucial aspects of memory function, neuronal activity, and neuroinflammation. These physical and functional interactions of BRI2 with AD‐linked genes, such as APP and TREM2, and its roles in pathways related to memory function, neuronal activity, and neuroinflammation suggest that the possibility of loss of BRI2 function contributing to the development of dementia is not far‐fetched.

Alzforum, a prominent scientific reference platform in AD/ADRD research, lists only two accepted models of FDD, and both are TG models with amyloid deposition (the ADanPP and the Tg‐FDD) explaining their acceptance as mouse models of FDD. The ADanPP transgenic mice overexpress a human BRI2 minigene carrying the FDD mutation, driven by the Syrian hamster prion protein promoter (Coomaraswamy *et al*, 2010) and exhibit ADan deposition, which is first observed at 2 months of age and increases with advancing age. However, human and rodent *ITM2B* expression in the CNS is highest in microglia. The use of the Syrian hamster prion protein promoter, which directs TG expression in neurons, could disrupt this natural expression pattern, potentially affecting the model's ability to accurately replicate the pathogenic mechanisms of the FDD mutation in different CNS cell types. Furthermore, this approach increases mature BRI2 protein levels (Coomaraswamy *et al*, 2010), from the existing endogenous WT *Itm2b* mouse alleles and from the TG‐FDD mutant alleles that will add to the pool of mature BRI2 protein even if the mutation reduces the efficiency of BRI2 maturation. Interestingly, but not surprisingly, when ADanPP mice were crossed with Aβ‐depositing APP‐PS1 TG mice, double‐TG ADanPP/APP‐PS1 mice exhibited a reduction in Aβ deposition compared with APP‐PS1 single‐TG littermates (Coomaraswamy *et al*, 2010), despite FDD patients showing co‐deposition of Aβ and ADan. This seemingly counterintuitive result in ADanPP mice echoes previous observations in mice overexpressing wild‐type BRI2 (TG‐BRI2). Double‐TG BRI2/CRND8 mice, resulting from crossing TG‐BRI2 with the Aβ‐depositing TG APP model CRND8 mice, exhibited reduced Aβ deposition compared with CRND8 TG littermates (Matsuda *et al*, 2008). These results align with the dual antiamyloidogenic function of mature BRI2, which is upregulated in both ADanPP and TG‐BRI2 models, involving reductions in both Aβ production through interactions with APP (Matsuda *et al*, 2008) and Aβ aggregation via the BRICHOS domain of BRI2 (Tambaro *et al*, 2017; Chen *et al*, 2020). Indeed, if the FDD mutation acts as a hypomorphic mutation in humans, causing a loss of mature BRI2 function that contributes to the disease, then the transgenic model, which shows a gain of mature BRI2 function, would represent the exact opposite of the disease's underlying mechanism. No information is available regarding the specific location of the transgene insertion in this model.

In essence, KI models are constructed based on objective, well‐established human genetic evidence, ensuring accurate representation of the genetic changes associated with AD/ADRD. They allow for an unbiased and “naïve” analysis of potential pathogenic pathways, considering both the central amyloid cascade hypothesis, when built on a genetic background expressing humanized *App* and human Aβ in a physiological manner (see below), and alternative or concurrent pathways. These models also allow exploration of the possibility that AD/ADRD‐linked genes encode proteins with functional implications in learning and memory and that pathogenic mutations/variants may alter these functions, ultimately leading to neurodegeneration via reduced expression or functional alterations mechanisms. This approach can lead to the discovery of previously unforeseen pathogenic pathways, identification of novel therapeutic targets, and development of therapeutic compounds targeting these pathways.

While the arguments supporting the KI gene‐editing approach over the TG method for modeling AD/ADRD in rodents are strong, a key question persists: Can the mouse and/or rat brain accurately replicate the pathogenic pathways triggered by AD/ADRD‐linked mutations in humans? Regardless of whether Aβ peptides and/or NFTs have a primary role in AD pathogenesis, it is important for model organisms to replicate these molecules in a manner that closely mirrors human physiopathology. A challenge in modeling AD in mice and rats arises from the 3‐amino acid difference between rodent and human Aβ. Since aggregated forms of Aβ are central to AD pathology, and human Aβ is more prone to forming toxic species than rodent Aβ, KI rodent models cannot properly test the amyloid cascade hypothesis. To address this, KI mice and rats with humanized *App* alleles, producing human Aβ instead of rodent Aβ in a physiologically relevant manner, have been generated (Tambini *et al*, 2019; Serneels *et al*, 2020; Baglietto‐Vargas *et al*, 2021). Humanized *App* rodents provide a platform to investigate the influence of human Aβ on AD pathogenesis and to study mutations in AD/ADRD‐related genes (e.g., *APP*, *PSEN1*, *TREM2*, and *ITM2B*) in the presence of physiologically relevant human Aβ. Regarding tau, there is a notable distinction between mice and rats. The human adult brain presents six tau isoforms, characterized by the presence or absence of two 29‐amino acid amino‐terminal inserts designated as 0N, 1N, or 2N tau, as well as the presence or absence of the R2 domain—one of tau's four microtubule‐binding regions. Isoforms containing the R2 domain are termed 4R, whereas those lacking it are known as 3R isoforms. While adult mouse brains express solely the three 4R tau isoforms (McMillan *et al*, 2008), adult rat brains express both 3R and 4R tau isoforms, akin to the human adult brain (Hanes *et al*, 2009). Hence, rats with humanized *App* alleles expressing human Aβ and human‐like tau isoforms can better model dementias characterized by both tauopathy and amyloidosis than mice. Yet, the potential negative impact of unknown human‐rodent brain differences on the effectiveness of rodents in AD/ADRD modeling, regardless of gene‐editing techniques, remains a valid consideration.

How can we evaluate the accuracy of KI lines in mimicking AD/ADRD without the presence of typical pathology? Is genetic alignment with the human disease sufficient to ensure faithful representation? Considering the potential influence of both chronological and biological age on AD pathology, model organisms with a lifespan of approximately 3 years might not be optimal for replicating AD pathology, even with *App* humanization. Consequently, we can seek proof‐of‐concept evidence that KI pathogenic mutations have pro‐amyloidogenic properties in the rodent brain. For example, humanized *App* KI rats carrying either the pathogenic *APP* Swedish mutation or the *PSEN1* mutation (*PSEN1L435F*) do not form plaques, whereas rats harboring both mutations exhibit amyloid plaques that replicate the distribution, characteristics, and Aβ‐species composition amyloid plaques observed in AD patients (Tambini *et al*, 2023). These dual mutant rats meet the criterion for pathology validation, establishing them as validated models of AD. Additionally, this observation underscores the inherent pro‐amyloidogenic nature of each mutation within the rat brain, suggesting that rats with singular mutations could potentially develop AD‐like pathology if their lifespan were extended. This evidence lends support to the use of single *APP* Swedish and *PSEN1L435F* KI mutant rats, accurately resembling the human disease genotype, as appropriate model organisms of AD.

Additional validation criteria could include the emergence of other alterations mirroring those observed in AD, such as neuroinflammation, astrogliosis, neuronal and synaptic loss, along with increased CNS levels of phosphor‐tau (NFT precursor) and Aβ species (amyloid plaque precursor). Furthermore, the identification of AD‐like molecular changes, such as transcriptomic profiles, could aid in validating KI models of AD. Lastly, a crucial element of validation is the appearance of clinical AD manifestations, especially cognitive decline. Essentially, with human therapeutic efforts focused on reversing, halting, or slowing cognitive decline, ideal animal models for testing drug candidates in preclinical trials should exhibit cognitive decline triggered by mechanisms mirroring those implicated in AD.

In conclusion, though the suitability of rodents as AD/ADRD model organisms is still not definitively established, it is reasonable to anticipate that humanized *App* KI rodent models, particularly rats, could offer a more efficient platform for uncovering pathogenic mechanisms, pinpointing potential targets for drug development, and assessing the therapeutic effectiveness of candidates in preclinical trials. This approach holds the potential for enhanced translatability compared with TG models, which introduce confounding effects since they are designed to fit a preconceived pathogenic model. While it is often stated that no AD/ADRD model is perfect and each has its strengths and weaknesses, it is also true that models could be categorized into three groups: the good, the bad, and the ugly, based on their overall quality and suitability for their intended purposes.

## Author contributions

**Luciano D'Adamio:** Conceptualization; funding acquisition; writing – original draft; writing – review and editing.

## Disclosure and competing interests statement

The author declares that he has no conflict of interest.
